# Feasibility evaluation of a dual-mode ankle exoskeleton to assist and restore community ambulation in older adults

**DOI:** 10.1017/wtc.2022.12

**Published:** 2022-07-01

**Authors:** Ying Fang, Karl Harshe, Jason R. Franz, Zachary F. Lerner

**Affiliations:** 1 Department of Mechanical Engineering, Northern Arizona University, Flagstaff, Arizona, USA; 2 Joint Department of Biomedical Engineering, University of North Carolina at Chapel Hill and North Carolina State University, Chapel Hill, North Carolina, USA; 3 College of Medicine – Phoenix, University of Arizona, Phoenix, Arizona, USA

**Keywords:** ankle, balance, elderly, exoskeleton, mobility, rehabilitation

## Abstract

**Background:**

Age-related deficits in plantar flexor muscle function during the push-off phase of walking likely contribute to the decline in mobility that affects many older adults. New mobility aids and/or functional training interventions may help slow or prevent ambulatory decline in the elderly.

**Objective:**

The overarching objective of this study was to explore the feasibility of using an untethered, dual-mode ankle exoskeleton as a mobility aid to reduce energy consumption, and as a resistive gait training tool to facilitate functional recruitment of the plantar flexor muscles.

**Methods:**

We recruited six older adults (68–83 years old) to evaluate acute metabolic and neuromuscular adaption to ankle exoskeleton assistance and to evaluate the potential for ankle resistance with biofeedback to facilitate utilization of the ankle plantar flexors. We also conducted a 12-session ankle resistance training protocol with one pilot participant.

**Results:**

Participants reached the lowest net metabolic power and soleus integrated electromyography (iEMG) at 6.6 ± 1.6 and 5.8 ± 4.9 min, respectively, during the 30-min exoskeleton assistance adaptation trial. Four of five participants exhibited a reduction (up to 19%) in metabolic power during walking with assistance. Resistance increased stance-phase soleus iEMG by 18–186% and stance-phase average positive ankle power by 9–88%. Following ankle resistance gait training, the participant exhibited increased walking speed, endurance, and strength.

**Conclusions:**

Our results suggest that dual-mode ankle exoskeletons appear highly applicable to treating plantar flexor dysfunction in the elderly, with assistance holding potential as a mobility aid and resistance holding potential as a functional gait training tool.

## Introduction

Preventing walking disability in our aging population is an enormous public health challenge. Mobility impairment is pervasive among older adults; 17, 28, and 47% of people in the United States aged 65–74, 75–84, and 85+ years, respectively, reported that difficulty in walking interferes with their daily activities (Greenberg and Fowles, 2011). Fundamental to their loss of independent mobility is that older adults walk more slowly, with shorter steps, and have higher metabolic energy costs than young adults—factors that have a severe negative impact on quality of life (Daly et al., 2013). These walking impairments are governed in large part by reduced force-generating capacity and functional output from muscle-tendon units spanning the ankle, resulting in reduced ankle power output via the plantar flexor (i.e., calf) muscles the during the propulsive “push-off” phase of walking (Franz, 2016; Boyer et al., 2017).

Age-associated deficits in ankle push-off during walking are highly resistant to conventional intervention; interventions designed solely to strengthen the plantar flexor muscles have generally failed to improve push-off power or walking economy. Isolated strengthening interventions seem to be unable to facilitate transfer between improved muscle force-generating capacity and more enhanced ankle push-off, and therefore do not change global measures of gait performance (Berg and Lapp, 1998; Beijersbergen et al., 2013, 2017). We suspect that these disappointing translational outcomes from isolated muscle strengthening arise from poor task-specificity in the context of walking. There appears to be a neuromuscular disconnect between newfound strength gains and functional utilization of muscle during walking, which motivates the need for new, yet accessible interventions capable of training improved plantar flexor utilization during walking in the elderly.

Lower-limb exoskeleton assistance targeting the ankle joint may hold potential for augmenting bouts of high-intensity ambulatory activities in older adults by reducing metabolic energy cost (Galle et al., 2017; Cseke et al., 2022). However, we are not aware of any research that has been conducted using an untethered powered exoskeleton, which limits the relevance of prior findings when evaluating the potential for battery-powered wearable robotic technologies to eventually become adopted as a mobility aid in the community. Additionally, little-to-no published work has quantified the time-course muscle activity responses to ankle exoskeleton assistance in older adults. Insight into how the neuromuscular system of older adults adapts to assistance may improve our ability to design intervention studies and anticipate long-term effects.

Conversely, wearable robotic systems could conceivably fill gaps in existing interventions used to treat age-related walking decline. For example, resistive exoskeletons targeting ankle plantar flexor engagement during push-off could improve the effectiveness of functional gait training and help meet the demand to provide therapy to our aging population. Wearable robotic resistance has yet to be explored in this population. However, considering the mechanistic link between plantar flexor dysfunction and walking ability limitations, the feasibility of using targeted ankle resistance to facilitate plantar flexor muscle recruitment and ankle power is clinically important. Research is needed to determine how willing or resistant older individuals would be to engage with relatively complex wearable robotic technologies.

Our overarching objective was to explore the feasibility of using a dual-mode ankle exoskeleton for treating walking disability in the elderly; testing the device in assistance mode as a mobility aid to reduce energy consumption, and as a resistive gait training tool to facilitate functional recruitment of the plantar flexor muscles. The specific goals of this feasibility study were twofold. First, we sought to quantify acute metabolic and neuromuscular adaption to ankle exoskeleton assistance during walking in older adults. We also sought to investigate if individuals with signs of age-related declines in walking function, as measured by a higher walking energy cost, would benefit more from ankle exoskeleton assistance compared to older adults that walk more efficiently. We hypothesized that older individuals would accommodate to ankle exoskeleton assistance within 20 min, as evidenced by consistent muscle activity patterns, and that age-related declines in walking efficiency would be associated with greater improvements in assisted walking economy relative to walking without the device. Second, we sought to validate the potential for push-off phase ankle resistance to facilitate functional utilization of the ankle plantar flexors during walking. We hypothesized that the ankle resistance mode, which combines plantar flexor resistance with real-time plantar pressure biofeedback, would significantly increase push-off phase plantar flexor muscle activity during use in older adults. This work will lay the foundation for the design of longer-term robotic gait training studies aimed at improving mobility in the elderly.

## Methods

This study utilized an untethered ankle exoskeleton that can operate to provide ankle plantar flexor assistance as a mobility aid and plantar flexor resistance as a functional muscle recruitment training platform. We developed protocols to evaluate the feasibility and efficacy of both assistance and resistance modes in a small cohort of elderly participants over two separate visits. Each protocol reflected how we envision these interventions may be used in future practice in this population. For assistance mode, ankle assistance may be used to augment prolonged bouts of ambulatory activity, so we sought to understand how older adults would adapt to assistance over a continuous 30-min interval. Resistance mode, on the other hand, was designed for short, high-intensity bouts of functional muscle recruitment training, so our resistance protocol was designed to test this mode over repeated 1-min trials. Lastly, one pilot participant completed a 12-session ankle resistance training protocol and pre/post assessments.

### Dual-Mode Exoskeleton

**Figure 1.**
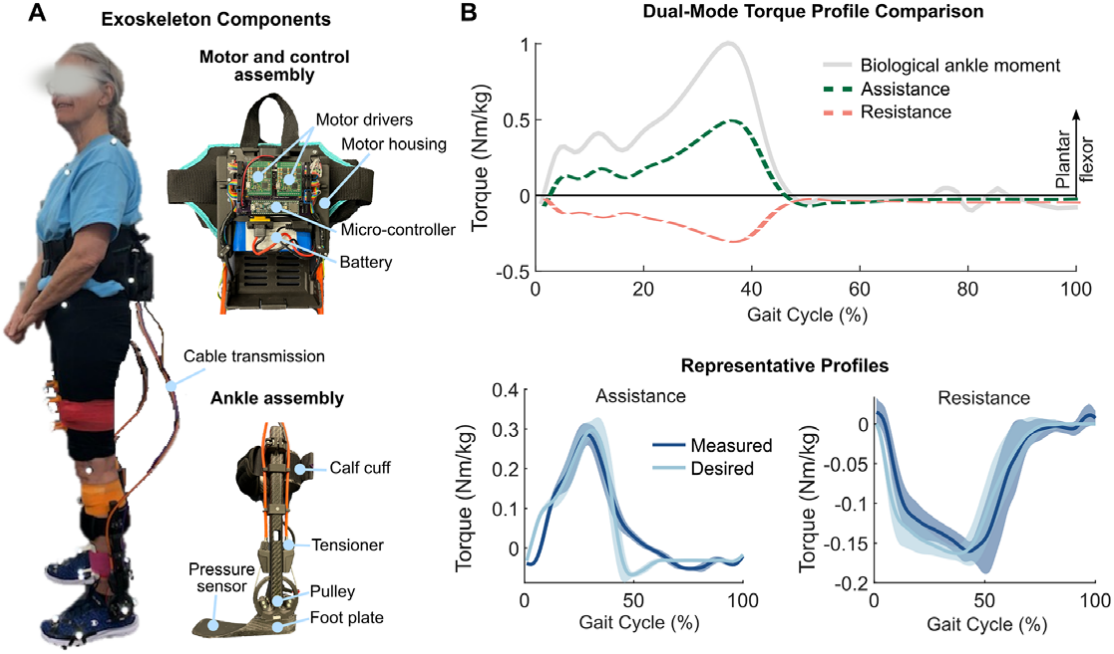
(a) Pictures of the exoskeleton on a participant with detailed views of the exoskeleton waist and ankle assemblies. (b) Example torque profiles for dual-mode assistance and resistance relative to the biological ankle moment.

The dual-mode ankle exoskeleton was battery-powered and utilized Bluetooth communication for wireless control. For actuation, we utilized Maxon brushless DC motors (EC-4pole) to bidirectionally actuate a pulley at each ankle joint via a chain-to-cable transmission system. To minimize distal mass, the motors, battery, and electronics were located in an assembly worn at the low back. The exoskeleton’s ankle joint included a custom torque sensor for low-level closed-loop torque-feedback control. Torque was transmitted to the body from each pulley to carbon fiber calf cuffs and foot plates. Tekscan embedded fore-foot pressure sensors (A502) were used to identify stance and swing phase of a gait cycle, and inform the assistive and resistive high-level control strategies (Figure 1a). Additional details of the design can be found in our prior publication (Orekhov et al., 2021).

We used an adaptive control strategy to provide torque profiles that were proportional to the real-time estimates of the biological ankle moment during stance phase; when operated in assistance mode, the controller specified plantar flexor directed torque as a percent of the biological ankle moment, and when operated in resistance mode, the controller specified dorsiflexor directed torque to match the inverse of the biological moment (Figure 1b). The torque profiles, generated through calibration and measurement of the fore-foot pressure sensors, inherently matched each individual’s walking pattern and walking speed, contributing to a seamless interaction between the device and its user. The adaptive controller was previously validated and achieved >90% accuracy in estimating biological ankle moment during level walking when providing assistance (Gasparri et al., 2019; Bishe et al., 2021) and resistance (Conner et al., 2020). Under assistance mode, we provided bilateral stance-phase plantar flexor assistance that reached a peak of 0.3–0.35 Nm/kg and swing-phase dorsiflexion assistance of 0.02–0.03 Nm/kg; assistance was adjusted between these ranges based on user feedback. Under resistance mode, we provided bilateral stance-phase plantar-flexor resistance that reached a peak of 0.15 Nm/kg; no resistance was provided during swing. These torque magnitudes were informed from prior work in individuals with walking impairment (Conner et al., 2020; Fang et al., 2020).

**Figure 2.**
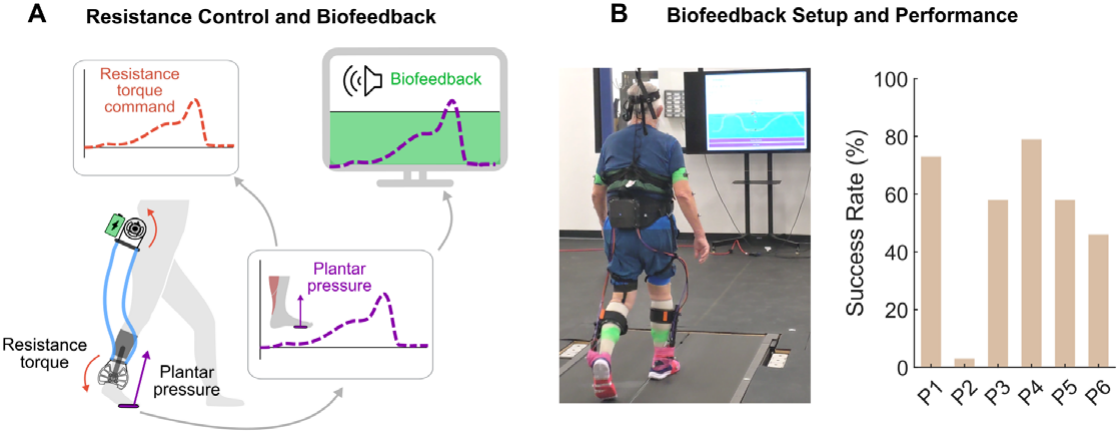
(a) Schematic depiction of the integrated exoskeleton and biofeedback system. (b) Picture of the biofeedback setup (left) and participant performance results (right).

To maximize user engagement, resistance mode also utilized an audio-visual biofeedback feature that provided real-time plantar pressure feedback (Conner et al., n.d.). Relying solely on embedded fore-foot sensors, biofeedback was incorporated within the exoskeleton system, and the user or operator can turn on and off the feature using the mobile application that controls the device. During the experiment, we displayed visual biofeedback on a TV in front of the treadmill. When plantar pressure reached the target threshold, the biofeedback line graph turned green, and a “ding” sound was emitted (Figure 2a). The threshold was set as 1.1 times the plantar pressure measured for each participant’s walking pattern without biofeedback or resistance; using a setpoint above 1.0 was used to motivate plantar flexor muscle activation well-above baseline.

### Participants

The study was approved by the Institutional Review Board of Northern Arizona University (NAU) under protocol #986744-27 on March 12, 2020. All participants read and signed an informed consent form prior to starting the study. Six healthy individuals between 68 and 83 years of age participated in this study (Table 1). Inclusion criteria included: (a) age between 65 and 85 years, and (b) self-reported ability to walk continuously for 30 min on a treadmill. Exclusion criteria included: (a) a history of dizziness and imbalance, (b) cardiovascular, respiratory, neurological, and musculoskeletal abnormalities as well as any other difficulties hindering normal gait, and (c) preexisting lower extremity pathologies such as chronic ankle instability, severe osteoarthritis, or joint replacement. The first five participants completed the Modifiable Activity Questionnaire that reflects their activity level for the past year (Eaglehouse et al., 2016), and completed both assistance and resistance protocol. The last participant (P6) completed the resistance protocol and a 12-session resistance training protocol.

**Table 1.** Participant details

Participant	Age	Sex	Mass (kg)	Height (m)	Walking speed (m/s)	Activity level (hours/week)
1	68	F	64	1.56	1.4	5.4
2	68	F	62.7	1.67	1.2	4.4
3	73	F	66.4	1.60	1.2	8.4
4	81	M	74.8	1.70	1.2	1.8
5	81	M	71.9	1.67	1.15	1
6	83	M	64.4	1.59	—	—

### Assistance Protocol

On the first visit, we fitted the device and determined each participant’s preferred moderate-to-fast walking speed that fell within the dimensionless walking speed range of 0.4–0.5 (Moissenet et al., 2019). Participants then completed two treadmill walking conditions with at least 20-min rest in between: (a) Exo-Adaptation: 30-min walk with the device as it provided bilateral plantarflexion and dorsiflexion assistance and (b) Shod: 6-min walk wearing shoes and without the device. We collected O_2_ and CO_2_ volumetric rates using a portable metabolic system (K5, Cosmed) and bilateral soleus activity using a wireless electromyography (EMG) system (1,926 Hz; Trigno, Delsys) throughout the entire trial for the two conditions. We also collected lower limb kinematic data using ten infrared motion capture cameras (120 Hz; Vicon Motion Systems, Oxford, UK), kinetic data from an instrumented treadmill (960 Hz; Bertec, Columbus, OH), and bilateral muscle activities at the tibialis anterior, vastus lateralis, and semitendinosus. Prior to the Exo-Adaptation trial, all participants had minimum experience (<1 min) walking with the exoskeleton within the past month.

### Resistance Protocol

On the second visit, participants walked under the following conditions: (a) Baseline: walking wearing shoes without the device, (b) Resisted: walking with bilateral plantar flexor resistance and biofeedback provided to the right leg. Participants did not have any exposure to resistance or biofeedback before the test. Biofeedback was provided unilaterally during this protocol to make it easier for participants to focus on a single limb. A set speed of 1 m/s was used for all participants except for P6, who walked at 0.75 m/s due to functional limitations. To minimize learning and ordering effects, all participants completed two 1-min trials following the order Baseline, Resisted, Resisted, Baseline; at least 2 min of rest was provided between conditions. We collected lower limb kinematic data using ten infrared motion capture cameras (120 Hz; Vicon Motion Systems, Oxford, UK), kinetic data from an instrumented treadmill (960 Hz; Bertec, Columbus, OH), and bilateral soleus activity using a wireless EMG system, for the entire 1-min of each trial.

### Resistance Training Protocol

One participant (P6) completed 12 sessions of resistance training over 4 weeks. Each session included a total of 20 min of walking with resistance and biofeedback on a treadmill; frequent rest breaks were provided throughout each visit. The first session began with the resistance level set at 0.15 Nm/kg and the treadmill speed set at 0.75 m/s; these values increased throughout the training and reached 0.23 Nm/kg and 1 m/s, respectively, in the last session (Figure 6). The participant completed pre- and post-assessments where we measured: plantar flexor strength during a maximum voluntary contraction using a handheld dynamometer (microFET2, Hoggan Scientific, Salt Lake City, UT), self-selected and fast walking speeds as the average of three bouts of 30-m walk, 6-min walk test (6MWT) distance, and metabolic power during a 6-min treadmill walk at a set speed (1.1 m/s).

**Figure 3.**
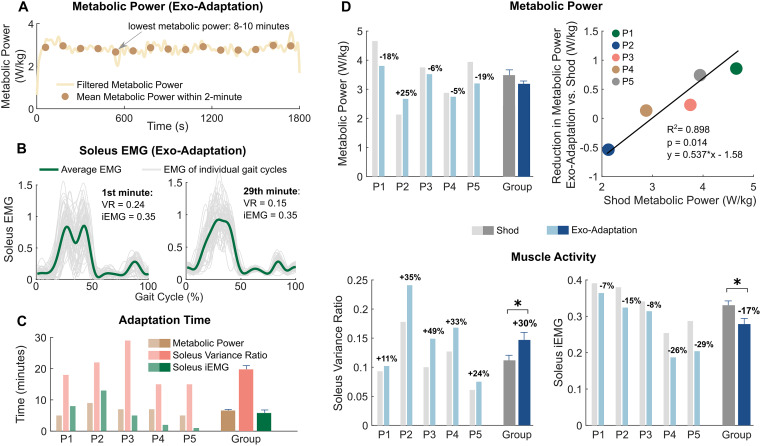
(a) Representative metabolic power measurements across a 30-min adaptation trial. (b) Representative soleus activity curves of all gait cycles (gray) and the average soleus activity curve (green) during the 1st and 29th minute of the 30-min adaptation trial. (c) Time to minimum metabolic power, soleus variance ratio, and soleus iEMG of each individual and the group. (d) Minimum metabolic cost, soleus variance ratio, and soleus iEMG during the 30-min adaptation trial compared to the fourth—sixth minute average of the shod trial for each individual and the group. Asterisks indicate statistical difference with *p* < .05.

**Figure 4.**
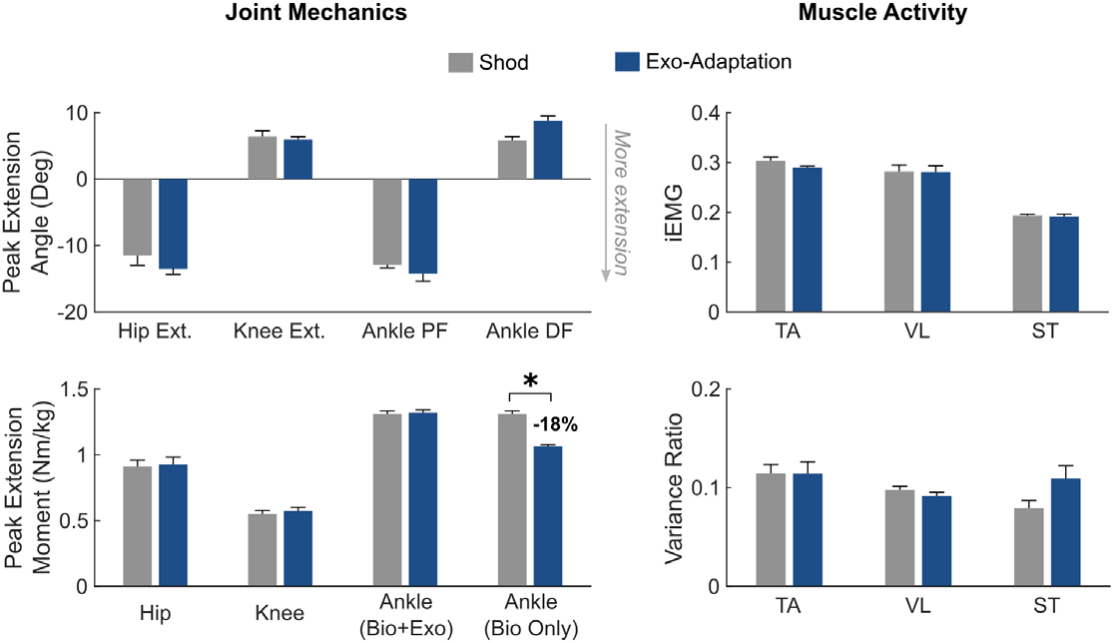
A comparison of group-level peak hip and knee extension angles and moments, peak ankle plantarflexion (PF) and dorsiflexion (DF) angle, peak ankle total moment (biological + exoskeleton) and biological moment, and iEMG and variance ratio of the tibialis anterior (TA), vastus lateralis (VL), and semitendinosus (ST) during the minute from each participant’s 30-min adaptation trial with the lowest metabolic power compared to the fifth—sixth minute average of the shod trial. Asterisks indicate statistical difference with *p* < .05.

**Figure 5.**
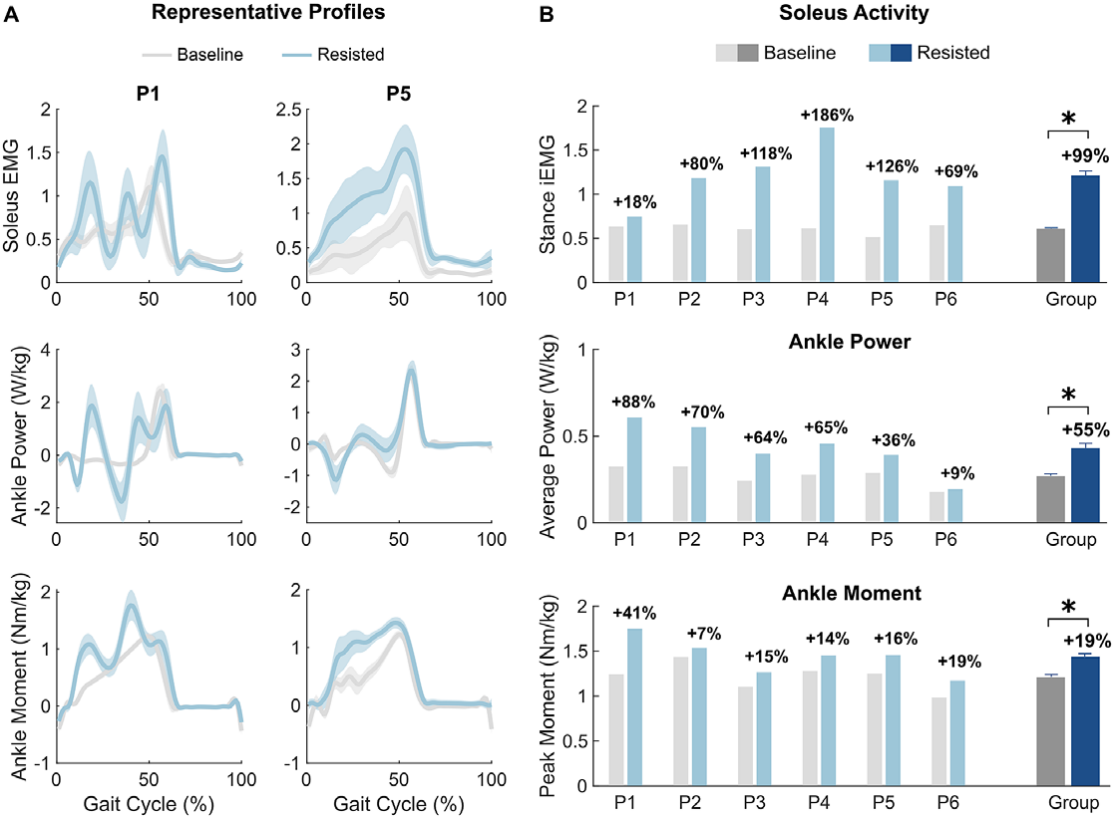
(a) Representative soleus activation, ankle power, and ankle moment profiles during baseline (shod) and Resisted walking conditions; multimodal (left) and unimodal (right) responses are shown. (b) Stance-phase soleus iEMG, average positive ankle power, and peak ankle plantarflexion moment for each participant and the group during baseline (shod) and Resisted walking conditions. Asterisks indicate statistical difference with *p* < .05.

**Figure 6.**
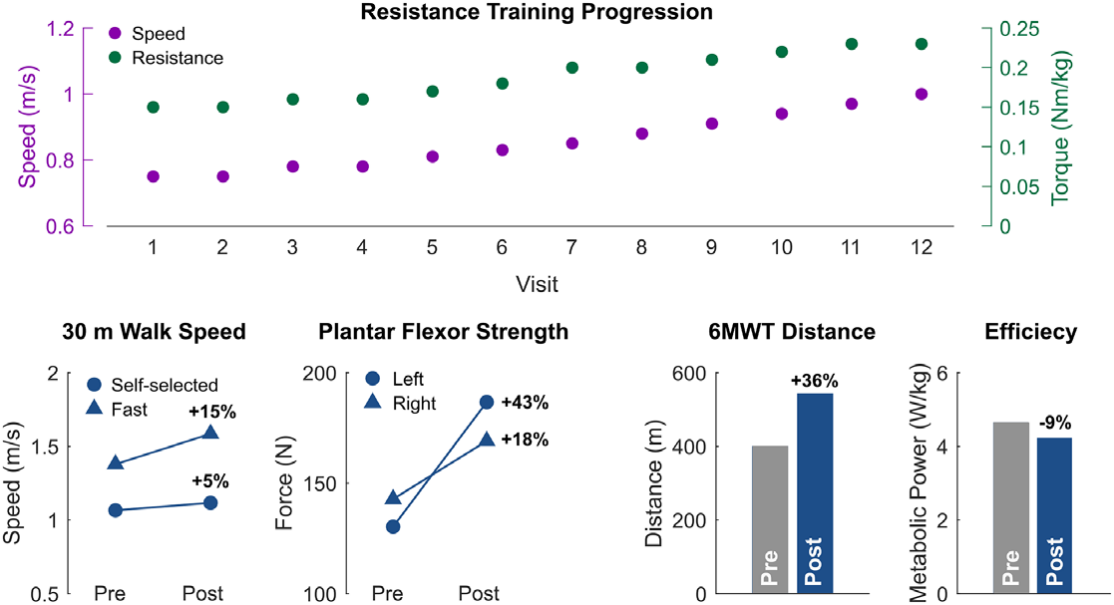
(Top) Walking speed and resistance torque across each training visit. (Bottom) self-selected and fast walking speeds, plantar flexor strength, 6-min walk test (6MWT) distance, and metabolic power during steady-state treadmill walking before (Pre) and after (Post) 12 sessions of resistance training for one participant (P6).

### Data Analysis

For the assistance visit, we analyzed metabolic data and muscle activity throughout the 30-min Exo-Adaptation trial and 6-min shod trial. Metabolic power was calculated from flow rates using Brockway’s standard equation (Brockway, 1987). Net metabolic power was obtained by subtracting the standing metabolic power from the metabolic power of each condition and divided by body mass, and then averaged across 2-min windows except for the first 4 min (i.e., 4–6 min and 6–8 min) (Galle et al., 2013; Poggensee and Collins, 2021). EMG data were band-pass filtered between 15 and 380 Hz, rectified, and low-pass filtered with a 7 Hz cutoff to generate the linear envelope. We normalized the filtered EMG signal by the peak value from walking with just shoes (Shod), and then segmented and normalized the data to percent gait cycle. We calculated integrated EMG (iEMG) by summing the area under the normalized EMG curve of the gait cycle and dividing by stride time.

We also calculated the variance ratio as in Bulea et al. (2018), which reflects the stride-to-stride repeatability of muscle activity, using the following equation:
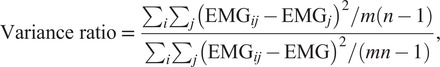
where *i* = 1 … *m* is the number of gait cycles, *j* = 1 … *n* is the time points within each gait cycle, EMG*_ij_* is the EMG of gait cycle *i* at time point *j*, EMG*_j_* is the mean EMG at time point *j* across all gait cycles, and EMG is the mean EMG across all gait cycles and time points. Variance ratio ranges from 0 to 1, with larger values indicating higher stride-to-stride variability. By capturing how repeatable the muscle activity profile was across strides, this metric was computed as another way to assess acclimation to exoskeleton assistance. iEMG and variance ratio were averaged across 1-min windows (i.e., first minute, second minute, and third minute) (Gordon and Ferris, 2007) and across limbs for both trials and for all muscles. We identified the time needed to achieve minimum net metabolic power, soleus iEMG, and soleus variance ratio during the 30-min trial. Because the minimum metabolic power naturally occurred at the start of the trial, we determined the minimum value between the 4th and 30th minute for this measure. Finally, we compared joint mechanics during the minute with the lowest metabolic power of the Exo-Adaptation trial to that of the last minute (fifth–sixth minute) of the shod trial. We scaled a generic musculoskeletal model for each participant and computed joint angles and moments using the inverse kinematics and inverse dynamics analyses in OpenSim 3.3 (Delp et al., 2007). The biological ankle moment (muscle-tendon and ligament contributions) was calculated by subtracting the exoskeleton assistance from the total ankle moment (combined biological contribution and assistance torque) that was determined from inverse dynamics.

For the resistance visit, we analyzed ankle mechanics and muscle activity over the entire duration for each trial. Similar to assistance, we computed ankle angle and moment using the inverse kinematics and inverse dynamics analyses in OpenSim 3.3 (Delp et al., 2007). The biological ankle moment (muscle-tendon and ligament contributions) was found by adding the exoskeleton resistance to the total ankle moment (combined biological contribution and resistance torque) that was determined from inverse dynamics. Ankle power (W) was calculated as the product of the ankle moment (Nm) and the respective ankle angular velocity (rad/s). We calculated stance-phase average positive ankle power by integrating the positive area of the ankle power curve and dividing by stance duration. We used the same methods described above to process muscle activity data and calculate stance phase soleus iEMG. Outcome measures were averaged across all gait cycles within each trial and across two trials with the same condition. Our results reporting focused on the right leg, the limb for which biofeedback was provided.

### Statistical Analysis

We used paired two-tailed *t*-tests to compare net metabolic power, muscle iEMG and variance ratio, and joint angles and moments between the Exo-Adaptation and Shod conditions for the assistance protocol. For the resistance protocol, we compared stance phase soleus iEMG, stance-phase average positive ankle power, and peak ankle plantarflexion moment between the Shod and Resisted conditions. Kolmogorov–Smirnov tests were used to check the normality of the outcomes in each comparison. In an exploratory analysis, we used linear regression to assess the relationship between baseline metabolic power and change in metabolic power during Exo-Adaptation compared to Shod. A fixed significance level of *α* ≤ 0.05 was used for this feasibility study.

## Results

### Ankle Assistance

Participants reached the lowest net metabolic power, soleus variance ratio, and soleus iEMG at 6.6 ± 1.6, 19.8 ± 1.6, and 5.8 ± 4.9 min, respectively, during the 30-min Exo-Adaptation trial. Compared to Shod, the minimum soleus variance ratio was 30.3 ± 14.1% greater (*p* = .027) and the lowest soleus iEMG was 17.0 ± 10.2% (*p* = .009) lower for the Exo-Adaptation condition. There was no group-level difference in net metabolic power between Shod and Exo-Adaptation condition (*p* = .317), yet four of five participants exhibited a reduction (by up to 19%) in metabolic power during walking with assistance. There was a significant positive relationship between reduced metabolic power from walking with assistance during the exo-adaptation trial and the baseline net metabolic power during Shod; participants who had greater baseline metabolic power exhibited a greater reduction during the Exo-Adaptation condition (*R*^2^ = 0.898, *p* = .014) (Figure 3).

Walking with the exoskeleton reduced the peak biological ankle moment by 18.5 ± 5.1% (*p* = .003) compared to shod. There was no difference in peak ankle plantarflexion or dorsiflexion angle, or peak total ankle moment between two conditions. There were no statistically significant differences in peak extension angle or moment at the hip or knee, or iEMG and variance ratio of the tibialis anterior, vastus lateralis, or semitendinosus between two conditions (Figure 4).

### Ankle Resistance

Walking with resistance and biofeedback (Resisted condition) increased stance-phase soleus iEMG by 99.5 ± 57.2% (*p* = .007) compared to baseline. Additionally, stance-phase average positive ankle power and the peak ankle plantar flexor moment were 55.4 ± 28.3% (*p* = .013) and 18.6 ± 11.6% (*p* = .009) greater, respectively, during the Resisted condition compared to baseline (Figure 5). Our participants adopted two general strategies to increase their plantar flexor recruitment during resisted walking with biofeedback; four participants (P2, P3, P5, and P6) used one strategy that resulted in an increase in the unimodal push-off peak, while P1 and P4 exhibited a multimodal EMG profile (Figure 5).

Following the month-long resistance training program, the pilot participant’s self-selected and fast walking speeds increased from 1.07 to 1.12 m/s, and from 1.38 to 1.59 m/s, respectively; his 6MWT distance increased by 35% from 397 to 539 m; plantar flexor strength increased by 18 and 43% on the right and left sides, respectively; and treadmill walking metabolic power decreased by 9% compared to the preassessment (Figure 6).

## Discussion

The goals of this feasibility study were twofold. Our first objective was to quantify acute metabolic and neuromuscular adaption to ankle exoskeleton assistance during walking in older adults. We accept our hypothesis that older individuals would acclimate relatively quickly to ankle exoskeleton assistance, as evidenced by the time to reach minimums in metabolic power, muscle activity magnitude, and stride-to-stride variance. We also sought to answer the question: Do older adults with a higher metabolic cost of walking benefit more from ankle exoskeleton assistance? Our results provide initial evidence that greater age-related declines in walking efficiency may be associated with greater improvements in assisted walking economy, as demonstrated through the strong positive relationship between baseline walking metabolic power and improvement in exoskeleton-assisted metabolic power. Our second objective was to validate the potential for push-off phase ankle resistance to facilitate functional utilization of the ankle plantar flexors during walking. We accept our hypothesis that walking with plantar flexor resistance with real-time plantar pressure biofeedback would significantly increase push-off phase plantar flexor muscle activity during use in older adults.

Most previous studies find that metabolic cost of transport is higher in older versus younger adults, which may lead to reduced levels of community ambulation. The ankle is a logical target for lower-limb exoskeleton assistance intended to improve mobility in the elderly because ambulatory decline in this population is associated with plantar flexor muscle dysfunction. It is theorized that assistive exoskeletons might be attractive for older adults to use during discrete bouts of relatively high-intensity activities like hiking with family, or keeping up with grandchildren on the way to the park. Our results were similar to a prior study that found no group-level change in metabolic power relative to walking without wearing an exoskeleton (Galle et al., 2017). It is worth noting, however, that this prior study utilized a tethered ankle exoskeleton. Our finding of reduced metabolic power for four of five participants within 20 min of walking with untethered ankle assistance hints at the potential for utilizing wearable technology to aid higher-intensity real-world bouts of walking for some, but not all, older adults. We found that the two older adults in this cohort with greater baseline energy cost of walking realized walking efficiency benefits by up to 18–19%. While these preliminary results should be interpreted with caution, the participant-level findings reported here are the first evidence that untethered ankle exoskeleton assistance may hold potential to improve walking efficiency in the elderly. While additional individual improvements in walking efficiency might be observed with additional tuning or human-in-the-loop optimization of the assistive torque profiles (Poggensee and Collins, 2021), much additional research is necessary, including walking over ground and in real-world settings.

Older adult participants in this study did not appear to require longer acclimation to robotic assistance compared to results from younger individuals reported in past studies. Our participants seemed to have adapted to untethered ankle exoskeleton assistance relatively quickly, achieving their lowest metabolic power around the seventh minute of walking, which was less than the time reported for younger individuals to reach a minimum adapted metabolic state in a previous tethered ankle exoskeleton study (Galle et al., 2013). Similarly, it took our participants about 6 min to reach their lowest soleus iEMG, a value that was 17% lower than walking without the device. By comparison, Gordon and Ferris (2007) found that acclimation of soleus activation to tethered powered ankle exoskeleton assistance was 23.8 min. These comparisons to other studies that utilized different devices, control strategies, and relative walking speeds should be interpreted with caution. We expect that the relatively short adaptation time for our device could potentially be attributed to the adaptive nature of our controller and the moderate-to-fast walking speed.

The findings in the present study are aligned with what we found in a prior study with individuals with neurological impairment—after just 6 min of acclimation, adolescents with cerebral palsy had significantly reduced soleus activity and metabolic cost (Fang et al., 2021). Participant P2 was the only participant that did not exhibit a reduction in metabolic power when walking with assistance. A possible explanation for this lack of improvement for this participant was that her baseline walking speed did not result in a walking intensity suitable for obtaining a benefit from robotic assistance (i.e., the intensity was too low), as evidenced by her having a baseline metabolic power well below the rest of the cohort. This is consistent with evidence from our prior work that suggests users are more likely to receive a metabolic benefit if they are walking at a higher intensity or completing a higher intensity activity (Fang and Lerner, 2021; Fang et al., 2021). Compared to muscle activation magnitude and metabolic power, stride-to-stride repeatability of muscle activation profiles took longer to reach a minimum, and was always greater compared to Shod across the 30-min trial. This seems to suggest that continued acclimation is needed to reach near-shod soleus repeatability or that wearing an external device inherently introduces variability.

The improvement in ankle plantar-flexor recruitment contributed to a large (>50%) increase in average ankle power, suggesting that using the device in the resistance mode could be an effective functional strength training tool to slow or prevent muscle loss in elderly population (Papa et al., 2017). We observed two strategies being used to increase plantar pressure output during resisted walking with biofeedback: one relied on increasing the existing push-off peak, and the other relied on generating multiple peaks (Figure 5). While both strategies worked to increase muscle engagement and stance phase average positive ankle power, the former is likely more beneficial for gait training as it would reinforce a motor control pattern that leads to improved push-off. The latter strategy was likely a result of the fixed treadmill speed preventing increased forward propulsion for the participants that could exceed the resistive torque when meeting the biofeedback target, so we suggest allowing participants to increase their walking speed, if needed, when engaging with ankle exoskeleton resistance in future work.

Reduced ankle push-off power during walking is often associated with functional declines in mobility (Clark et al., 2013; Franz, 2016). The improvements in strength, 6MWT distance, and walking efficiency exhibited by the pilot training subject following the 12-session training protocol suggest that additional research on the use of functional gait training with wearable ankle resistance and biofeedback should be explored in a larger follow-on study. While a single-subject experiment should be interpreted with extreme caution, a strength of this work was that these results were obtained using a battery-powered wearable device and biofeedback system that could one day be used at home or in the community. Future home-based training interventions should explore the potential for this technology to improve long-term walking performance in this population.

The small cohort utilized for this study was an obvious limitation of this work. Prior to embarking on large-scale clinical investigations, our goal was to provide initial evidence that this technology holds potential in the elderly and that future work is warranted. We believe we were successful in this goal. Our current finding on the relationship between baseline metabolic rate and metabolic benefit from ankle assistance should be verified with a larger sample size. Future studies should increase the number of participants and evaluate individuals across a larger spectrum of age-related declines in mobility. Another limitation was that both assistance and resistance protocols were delivered on a single visit without follow-up or repeated testing. Exploring multiday adaptation with both assistance and resistance is necessary to inform implementation over extended time periods. Additionally, the short walking duration of the resisted trials was another limitation that leaves many open questions about duration of use required to elicit tissue remodeling. We expect that additional acclimation to assistance may demonstrate greater improvements in assisted walking efficiency. For resistance, we plan to conduct a training study similar to the pilot experiment reported here but with a full cohort of older adults.

## Conclusion

In conclusion, the results of this feasibility study suggest that dual-mode ankle exoskeletons may hold promise for treating plantar flexor dysfunction in some older adults, with assistance having potential as a mobility aid for acute high-intensity activities and resistance having potential as a functional gait training tool. By demonstrating that individuals with greater baseline metabolic power benefit more from powered ankle assistance, we can target these individuals for testing in future interventions. The increases in plantar flexor recruitment and average positive ankle power generation during walking with resistance, coupled with promising results from a single-subject training protocol, builds confidence for pursuing a multivisit gait training study. Our results reflect immediate changes with no prior practice, which suggests that ankle exoskeleton resistance with biofeedback is likely to be suitable for some older adults. By utilizing an untethered exoskeleton for this study, we demonstrate the potential for future interventions to take place at home or in community care settings. The insight gained from this study lays the foundation for the design of intervention studies aimed at improving community mobility and quality of life in the elderly.

## Data Availability

Please contact the corresponding author for data associated with this article. All reasonable requests will be honored.
